# Kenyon Cell Subtypes/Populations in the Honeybee Mushroom Bodies: Possible Function Based on Their Gene Expression Profiles, Differentiation, Possible Evolution, and Application of Genome Editing

**DOI:** 10.3389/fpsyg.2018.01717

**Published:** 2018-10-02

**Authors:** Shota Suenami, Satoyo Oya, Hiroki Kohno, Takeo Kubo

**Affiliations:** Department of Biological Sciences, Graduate School of Science, The University of Tokyo, Bunkyo-ku, Tokyo, Japan

**Keywords:** honeybee, hymenoptera, brain, mushroom body, Kenyon cell, learning and memory, genome editing

## Abstract

Mushroom bodies (MBs), a higher-order center in the honeybee brain, comprise some subtypes/populations of interneurons termed as Kenyon cells (KCs), which are distinguished by their cell body size and location in the MBs, as well as their gene expression profiles. Although the role of MBs in learning ability has been studied extensively in the honeybee, the roles of each KC subtype and their evolution in hymenopteran insects remain mostly unknown. This mini-review describes recent progress in the analysis of gene/protein expression profiles and possible functions of KC subtypes/populations in the honeybee. Especially, the discovery of novel KC subtypes/populations, the “middle-type KCs” and “KC population expressing FoxP,” necessitated a redefinition of the KC subtype/population. Analysis of the effects of inhibiting gene function in a KC subtype-preferential manner revealed the function of the gene product as well as of the KC subtype where it is expressed. Genes expressed in a KC subtype/population-preferential manner can be used to trace the differentiation of KC subtypes during the honeybee ontogeny and the possible evolution of KC subtypes in hymenopteran insects. Current findings suggest that the three KC subtypes are unique characteristics to the aculeate hymenopteran insects. Finally, prospects regarding future application of genome editing for the study of KC subtype functions in the honeybee are described. Genes expressed in a KC subtype-preferential manner can be good candidate target genes for genome editing, because they are likely related to highly advanced brain functions and some of them are dispensable for normal development and sexual maturation in honeybees.

The European honeybee (*Apis mellifera* L.) is a social insect (Winston, 1986; Seeley, 1995), and its colony members exhibit advanced learning abilities that can be relatively easily assayed using associative learning paradigms, even under laboratory conditions (Takada, 1961; Giurfa et al., 2001; Dyer et al., 2005; Hori et al., 2006, 2007). Therefore, the honeybee has long been used as a model animal for studying learning and memory in insects (Giurfa, 2007; Giurfa and Sandoz, 2012; Chittka, 2017).

Drafts of the honeybee whole genome sequence (Honeybee Genome Sequencing Consortium, 2006; Elsik et al., 2014) have greatly promoted studies of the honeybee molecular biology, neuroscience, and genetics. This mini-review focuses on a topic that has received little attention to date–the possible roles of KC subtypes that constitute the MBs, a higher-order center in the honeybee brain (Erber et al., 1980; Rybak and Menzel, 1998; Komischke et al., 2005; Locatelli et al., 2005; Menzel and Manz, 2005; Ito et al., 2008; Szyszka et al., 2008), and their possible evolution in hymenopteran insects.

## Unique gene/protein expression profiles of KC subtypes in the honeybee brain

### KC subtypes that constitute the honeybee mushroom bodies

**Figure 1:**
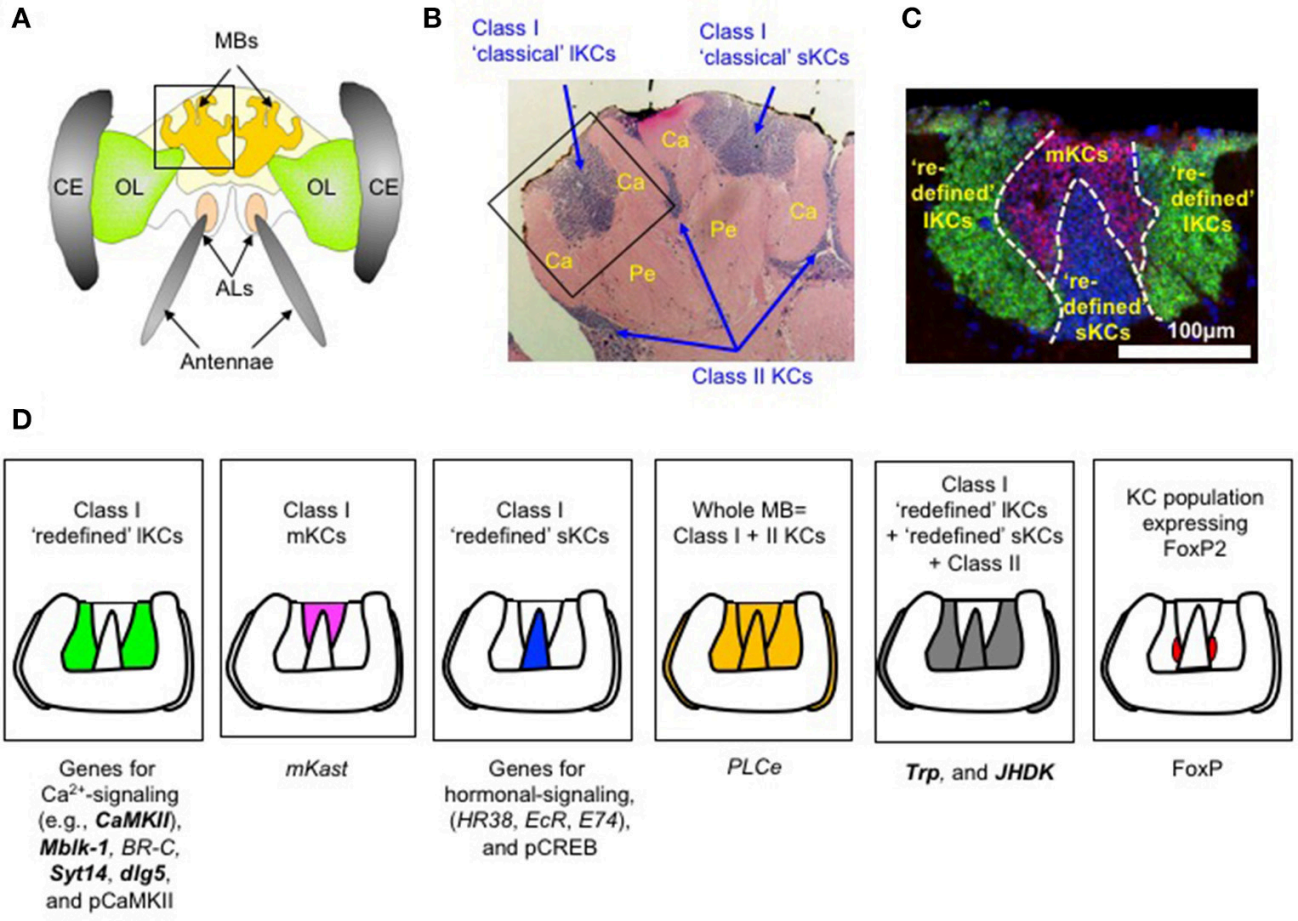
Example of genes and proteins expressed in a KC subtype-preferential manner in worker honeybee MBs. **(A)** Schematic drawing of the head and brain of a worker honeybee. MB, mushroom body; OL, optic lobe; AL, antennal lobe; CE, compound eyes. **(B)** Hematoxylin-eosin staining of a section of the left MB, which corresponds to the boxed region in **(A)**. Ca, calyx; Pe, pedunculus. Class I “classic” lKCs, “classic” sKCs, and class II KCs are indicated by arrows. **(C)** Double *in situ* hybridization of *CaMKII* (green), which is preferentially expressed in “redefined” lKCs, and *mKast* (magenta), which is preferentially expressed in mKCs in a single MB calyx. Redefined sKCs are stained with nuclear staining and colored blue. This picture well represents the presence of the three class I “redefined” KC subtypes: “redefined” lKCs, mKCs, and “redefined” sKCs. **(D)** Schematic drawing of five KC subtype-preferential gene expression patterns. Each box contains a schematic drawing of a single MB calyx, in which KC subtypes/populations with strong gene/protein expression are colored green (for class I “redefined” lKCs), magenta (for class I mKCs), blue (for class I “redefined” sKCs), yellow (for the whole MB = class I + II lKCs), gray (for class I “redefined” lKCs + “redefined” sKCs + class II KCs), and red (for KC population expressing FoxP). Genes with a KC subtype-preferential expression pattern discussed in this mini-review are listed below each box. Note that the genes whose expression in the “redefined” lKCs/ “redefined” sKCs was confirmed by double *in situ* hybridization with *mKast* are indicated by bold letters. These figures are cited from Kubo (2012) and Kaneko et al. (2016) with some modifications.

Several combinations of approaches including behavioral, pharmacological, electrophysiological, imaging, and ablation studies have revealed that mushroom bodies (MBs) play important roles in learning and memory, and sensory integration in the honeybee (Erber et al., 1980; Rybak and Menzel, 1998; Komischke et al., 2005; Locatelli et al., 2005; Menzel and Manz, 2005; Ito et al., 2008; Szyszka et al., 2008). In the honeybee, the MBs are a paired structure, each of which has two cuplike structures, called calyces, that are sensory input regions of the MBs (Figure 1A).

Honeybee MBs have long been thought to comprise three classes/subtypes of interneurons termed Kenyon cells (KCs): class I “classical” large- (lKCs or inner noncompact KCs) and “classical” small-type KCs (sKCs or inner compact KCs), and class II KCs (or outer compact KCs), which are distinguished by their cell body size and location in the MBs (Figure 1B) (Mobbs, 1982; Strausfeld et al., 1998; Strausfeld, 2002; Farris et al., 2004; Farris, 2005; Fahrbach, 2006). The somata of “classical” class I lKCs are located at the inside edges of the MB calyces, whereas those of “classical” sKCs are located in the inner core of the MB calyces. The somata of class II KCs, on the contrary, are located at the outer surface of the MB calyces (Figure 1B) (Mobbs, 1982; Strausfeld et al., 1998; Strausfeld, 2002; Farris et al., 2004; Farris, 2005; Fahrbach, 2006). However, each of the “classical” lKCs projects its dendrites to the olfactory (lip) or visual (collar) subregions of the MB calyces, and the “classical” sKCs project their dendrites to the multimodal basal ring. Class II KCs project their dendrites to the entire calyx (Strausfeld, 2002; Farris et al., 2004).

Recently, Kaneko et al. (2013) identified the novel class I mKCs, which are characterized by the preferential expression of *middle-type-Kenyon cell-preferential arrestin-related protein* (*mKast*) (Figure 1C) (Kaneko et al., 2013). Therefore, the honeybee MBs actually comprise three subtypes of class I KCs: “redefined” lKCs, mKCs, and “redefined” sKCs. The somata of the mKCs are localized between the “redefined” lKCs and “redefined” sKCs, and the size of the somata of the mKCs is intermediate between the “redefined” lKCs and “redefined” sKCs (Figure 1C; Kaneko et al., 2013). Importantly, these KC subtypes exhibit differential gene expression profiles, suggesting they have distinct cellular characteristics and functions.

### lKCs

Honeybee MBs express more than 20 genes in a lKC subtype-preferential manner (for more comprehensive reviews, see Kubo, 2012; Kaneko et al., 2016). Among these genes, nine are expressed preferentially in the lKCs. Five of these 9 genes encode proteins involved in the intracellular Ca^2+^-signaling pathway, such as *Ca*^2+^*/calmodulin-dependent protein kinase II (CaMKII)* (Kamikouchi et al., 1998, 2000; Sen Sarma et al., 2009; Uno et al., 2012), which has an important role in the synaptic plasticity that underlies learning and memory abilities in various animals (Colbran and Brown, 2004; Elgersma et al., 2004; Pasch et al., 2011). Furthermore, Pasch et al. (2011) reported that phosphorylated (activated) CaMKII protein (pCaMKII) is present in lKCs, but not in sKCs or class II KCs (Pasch et al., 2011). These findings suggest that the lKCs are related to Ca^2+^-signaling-based learning and memory functions (Figure 1D; Ghosh and Greenberg, 1995; Rose and Konnerth, 2001; Perisse et al., 2009; Shonesy et al., 2014).

Matsumoto et al. (2014) used pharmacologic inhibition to indicate that CaMKII is involved in late long-term memory (LTM), but not in mid-term memory (MTM) or early LTM formation (Matsumoto et al., 2014). In addition, Scholl et al. (2015) used RNA interference (RNAi) and pharmacologic inhibition to indicate that CaMKII is necessary for both early and late LTM, but not for MTM (Scholl et al., 2015). Although the two studies reported different effects of CaMKII inhibition on early LTM, they consistently suggest that the lKCs play a role at least in late LTM formation in the honeybee.

Genes encoding for two transcription factors, *Mushroom body/large-type Kenyon cell-preferential gene-1* [(*Mblk-1*)/*E93*] (Takeuchi et al., 2001) and *Broad-Complex* (*BR-C*) (Paul et al., 2006), are also expressed preferentially in the lKCs in the honeybee MBs. The *MBR-1*, a nematode homolog of *Mblk-1*, is necessary for both pruning excessive neurites during development and learning ability (Kage et al., 2005; Hayashi et al., 2009). Thus, selective expression of *Mbk-1* in the lKCs is consistent with the speculation that synaptic plasticity is enhanced in the lKCs. It is also plausible that Mblk-1 and BR-C are involved in transactivation of genes expressed in an lKC-preferential manner in the honeybee brain.

Suenami et al. (2016) recently identified three genes, *synaptotagmin 14* (*Syt14*), *discs large 5* (*dlg5*), and *phospholipase C epsilon* (*PLCe*), whose expression is more highly enriched in the MBs of the honeybee brain than the previously identified KC subtype-preferential genes (Suenami et al., 2016). While, *Syt14* and *dlg5* are highly selectively expressed in the “redefined” lKCs in the MBs, *PLCe* is highly expressed in the whole MBs; i.e., all of the class I lKCs, mKCs, and sKCs and class II KCs (Figure 1D; Suenami et al., 2016). *Syt14* and *dlg5* are involved in membrane trafficking and spine formation, respectively (Fukuda, 2003; Hayashi et al., 2009; Doi et al., 2011; Wang et al., 2014; Suenami et al., 2016), implying that both synaptic transmission and synaptic plasticity are enhanced in the lKCs.

It is difficult to conclude definitely on the correspondence between the “classical” lKCs and “classical” sKCs, and “redefined” lKCs, mKCs, and “redefined” sKCs based on morphological observation. In some previous studies, which reported on genes expressed in a lKC-preferential manner, it seems that “classical” lKCs correspond to “redefined” lKCs, and “classical” sKCs correspond to mKCs plus “redefined” sKCs [for example, (Kamikouchi et al., 2000; Takeuchi et al., 2001; Uno et al., 2012)]. On the contrary, Strausfeld (2002) previously represented the boundary between the “classical” lKCs and “classical' sKCs, which are distinguished based on their morphology, just in the mKC area (Strausfeld, 2002). Therefore, future studies must investigate the actual correspondence between the “classical” lKCs and “classical” sKCs, and the “redefined” lKCs, mKCs, and “redefined” sKCs, by examining their gene expression profiles using double *in situ* hybridization with *mKast* (Kaneko et al., 2013).

### sKCs

Three genes, *ecdysone receptor* (*EcR*), *hormone receptor-like 38* (*HR38*), and *E74*, are expressed preferentially in the sKCs, and all of them encode transcription factors involved in the ecdysteroid-signaling pathway (Figure 1D; Paul et al., 2005; Yamazaki et al., 2006; Takeuchi et al., 2007). Expression of *HR38* is higher in the brains of foragers than in the brains of nurse bees, suggesting its possible association with the division of labor of workers (Yamazaki et al., 2006). The EcR/ultraspiracle (Usp) heterodimer binds to ecdysteroids to orchestrate transcriptional regulation during metamorphosis (Davis et al., 2005). In contrast, HR38 competes with EcR for Usp, and the HR38/Usp heterodimer activates the transcription of target genes distinct from those of the EcR/Usp heterodimer (Zhu et al., 2000; Baker et al., 2003). Thus, Yamazaki et al. (2006) previously proposed that the enhanced expression of *HR38* in the forager brain might contribute to switching the mode of ecdysteroid-signaling in the MBs from the EcR- to the HR38-mediated pathway in association with the division of labor of workers (Yamazaki et al., 2006).

Recent studies, however, reported that, in the silk moth and fruit fly, *HR38* is an immediate early gene, whose neuronal expression is activated by neuronal excitation (Fujita et al., 2013), and that *HR38* expression in the honeybee brain is induced by foraging behavior (Ugajin et al., 2018). These results suggest an alternative possibility that *HR38* expression in the sKCs of the honeybee brain is a consequence of the foraging behavior, and does not necessarily represent a gene expression profile specific to the forager brain. These possibilities need to be investigated further.

On the contrary, Gehring et al. (2016) reported that phosphorylated (activated) cAMP-response element binding protein (pCREB) is enriched in the sKCs in honeybee MBs (Figure 1D; Gehring et al., 2016), suggesting that the sKCs are related to CREB-based memory function (McGuire et al., 2005; Alberini, 2009; Hirano et al., 2016).

### mKCs

So far, only one gene, termed *mKast*, has been found to be expressed preferentially in the mKCs of the honeybee MBs (Figures 1C,D) (Kaneko et al., 2013). Although mKast belongs to the α-arrestin family, which is involved in downregulation of membrane receptors (Kaneko et al., 2013), the role of mKast in the honeybee is currently obscure. *mKast* expression in the brain begins at the late pupal stages and is detectable almost exclusively in the adult brain, suggesting its role in regulating adult honeybee behaviors and/or physiology (Yamane et al., 2017).

Since detection of neural activity using immediate early genes revealed that MB KCs (Singh et al., 2018; Ugajin et al., 2018), especially sKCs and some mKCs (Kaneko et al., 2013), are active in the brains of foragers, it is plausible that these KC subtypes are related to sensory information processing during the foraging flights.

### Broader gene expression profiles

Three genes, *PLCe* (Suenami et al., 2016), *protein kinase C* (*PKC*) (Kamikouchi et al., 2000), and *E75* (Paul et al., 2006), are preferentially expressed in all KC subtypes (=the whole MBs) in the honeybee brain (Figure 1D). Considering that *E75* is expressed preferentially in all KC subtypes (=the whole MBs) (Paul et al., 2006), whereas *EcR, HR38*, and *E74* are preferentially expressed in the sKCs (Paul et al., 2005; Yamazaki et al., 2006; Takeuchi et al., 2007), it might be that different ecdysteroid-signaling pathways function in distinct KC subtypes.

With regards to PLC, there are four homologs, including *PLCe*, in the honeybee. The *PLCe* is expressed almost selectively in the whole MBs, and expression of the other three homologs is significantly higher in the MBs than in other brain regions (Suenami et al., 2017). Suenami et al. (2017) revealed that pharmacological inhibition of PLC significantly attenuated the memory acquisition, but did not affect memory retention, suggesting that PLCs are involved in early memory formation in the honeybee (Suenami et al., 2017). Thus, although both CaMKII and PLC are involved in Ca^2+^-signaling (Smrcka et al., 2012; Dusaban and Brown, 2015), they play roles at different stages of learning and memory. It will be interesting to test whether their roles at different stages of learning and memory can be attributed to their distinct KC subtype-preferential expression.

Two genes, *tachykinin-related peptide* (*Trp*) and *juvenile hormone diol kinase* (*JHDK*), are preferentially expressed in both the “redefined” lKCs and “redefined” sKCs, but not in the mKCs (Figure 1D; Takeuchi et al., 2004; Uno et al., 2007; Kaneko et al., 2013). The Trps are multifunctional brain/gut peptides that have important roles in neurotransmission and/or neuromodulation (Van Loy et al., 2010). In *Drosophila*, tachykinin-expressing neurons control male-specific aggressive behaviors (Asahina et al., 2010). Therefore, it might be that *Trp* is also involved in the control of aggressive behaviors even in the honeybee. The function of JHDK in insects is not well understood (Uno et al., 2007).

Interestingly, McQuillan et al. (2012) reported that the expression of genes for amine receptors, which are involved in learning and memory, differs across KC subpopulations (McQuillan et al., 2012), which is consistent with the recent notion that different regions of the MBs contribute to learning and memory in *Drosophila* (Zars et al., 2001; McGuire et al., 2003; Trannoy et al., 2011).

### KC population expressing FoxP

Recently, Schatton and Scharff (2017); Schatton et al. (2018) identified a novel KC population expressing transcription factor FoxP in the MBs of the honeybee brain (Figure 1D) (Schatton and Scharff, 2017; Schatton et al., 2018). Although Kiya et al. (2008), who first reported the *FoxP* expression in the honeybee brain, detected no significant *FoxP* expression in the honeybee MBs (Kiya et al., 2008), Schatton et al. notified that, in *Drosophila*, a MB-core subpopulation expresses *FoxP*, which is related to decision-making (DasGupta et al., 2014). They also reported FoxP expression in the honeybee MBs (Schatton and Scharff, 2017). These findings suggest that neural populations with FoxP expression that are related to reinforcement-based learning abilities are conserved among animal species (Schatton and Scharff, 2017; Schatton et al., 2018).

There seems to be a problem, however: although Schatton et al. indicated that the KC population expressing FoxP does not overlap with mKCs, and speculated that FoxP specifies different subsets of mKC (Schatton and Scharff, 2017), Kaneko et al. (2013) and Suenami et al. (2016) reported that lKCs do not overlap with mKCs, and observed no gaps between the areas where lKC and mKC somata exist (Kaneko et al., 2013; Suenami et al., 2016). Based on the latter findings, the KC population expressing FoxP is assumed to be the lKCs. This point needs to be clarified in future studies.

## Analysis of KC subtype differentiation during metamorphosis

Genes expressed in a KC subtype-preferential manner can be used as markers to trace the differentiation of KC subtypes or their evolution in hymenopteran insects.

In honeybees, larval MBs comprise only class II KCs. Class I “classical” lKCs and sKCs are newly produced from proliferating neuroblasts whose somata are located in the inner core inside of the MB calyces during the pupal stages (Farris et al., 1999) and cease their proliferation at the P2 and P5 stages, respectively. Suenami et al. (2016) recently used three genes, *Syt14, dlg5*, and *PLCe*, as markers to trace the differentiation of the “redefined” lKC (*Syt14*, and *dlg5*) and all KC subtypes (*PLCe*) (Suenami et al., 2016). The *PLCe* is already expressed in larval MBs and continues to be expressed in the whole MBs during the pupal stages, suggesting that Ca^2+^-signaling is enhanced in the whole MBs during the entire honeybee lifespan. The expression of *Syt14* and *dlg5* becomes detectable at the middle pupal stages (around P3), and is restricted to the lKCs at the adult stage, suggesting that expression of *Syt14* and *dlg5* is characteristic of differentiated lKCs (Suenami et al., 2016). The FoxP expression is also not detected in larval MBs, but becomes detectable in the MBs at the middle-to-late pupal stages (P4-5) (Schatton et al., 2018), suggesting that FoxP expression is also characteristic of differentiated KCs. In contrast, KCs expressing *mKast* become detectable at the late pupal stages (P7 and P8) (Kaneko et al., 2013), suggesting that mKCs develop after the lKCs begin to differentiate or *mKast* is expressed at the late stage of mKC differentiation.

## Possible KC subtype evolution in hymenopteran insects

Farris and Schulmeister (2011) indicated that both aculeate insects and parasitic wasps, which are hymenopteran insects that appeared later in the course of evolution, have more morphologically elaborate MB calyces than sawflies, which are primitive hymenopteran insects, and proposed that the elaborate MB calyces are associated with the higher learning ability of parasitic wasps (Farris and Schulmeister, 2011). This leads to the question of when during the evolution of hymenopteran insects were KC subtypes acquired? To address this question, Oya et al. (2017) performed *in situ* hybridization of *Trp* homologs to compare KC subtypes among the brains of four hymenopteran insect species: (1) a phytophagous and solitary sawfly (Symphyta; *Arge similis*), (2) a solitary parasitic wasp (Apocrita; *Ascogaster reticulata*), (3) an eusocial hornet (Aculeata; *Vespa mandarinia*), and (4) a nidificating and solitary scoliid wasp (Aculeata; *Campsomeris prismatica*) (Oya et al., 2017). As *Trp* is expressed in both “redefined” lKCs and “redefined” sKCs, but not in mKCs; the presence of all three KC subtypes can be visualized in a certain hymenopteran insect brain by performing *in situ* hybridization of a single *Trp* homolog (Takeuchi et al., 2004).

**Figure 2:**
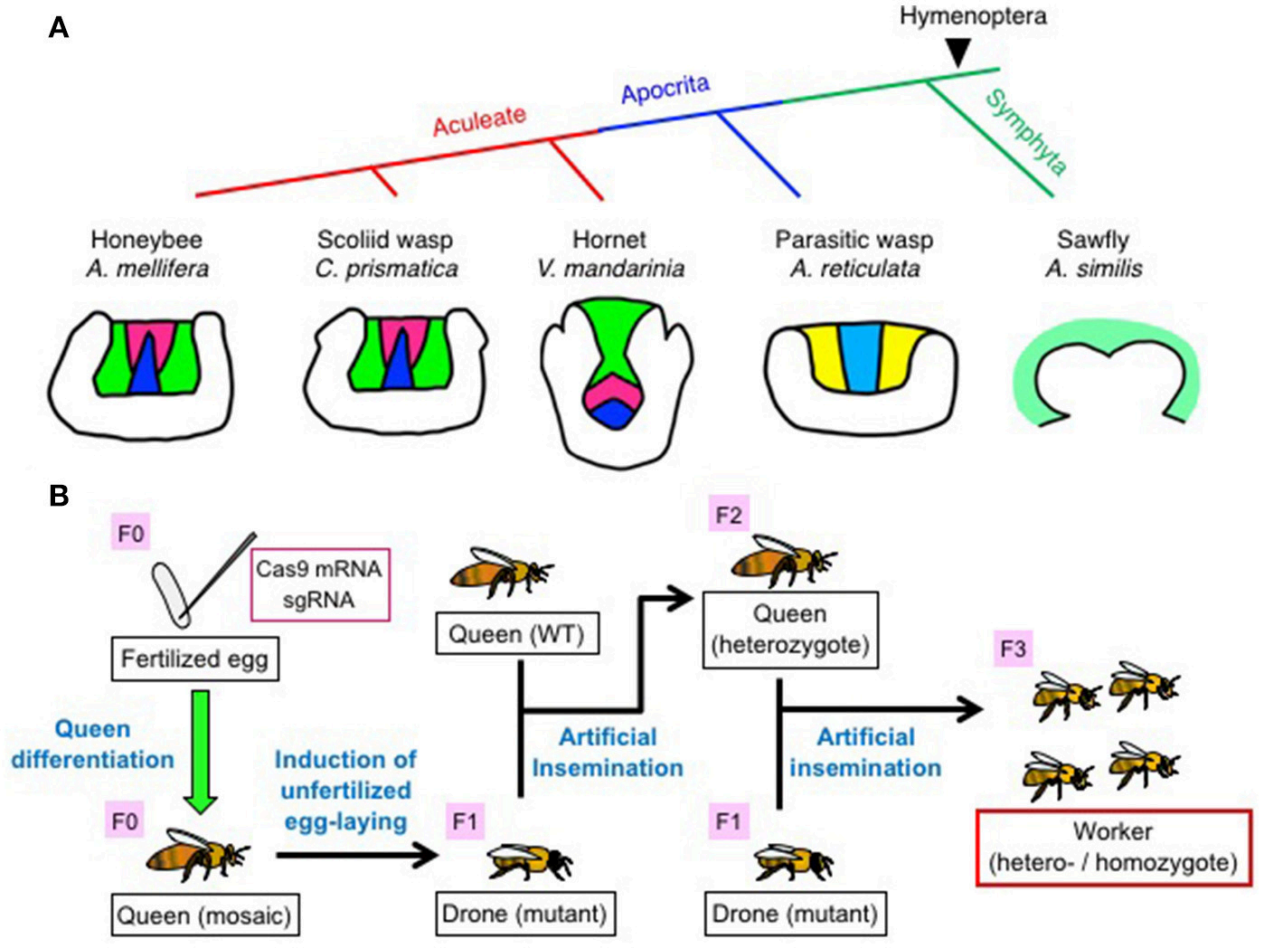
Comparison of KC subtypes in the MBs among various hymenopteran species **(A)** and flowchart for the production of mutant workers by genome editing **(B)**. **(A)** (Upper panel) Phylogenic trees of five hymenopteran species examined in the study (Oya et al., 2017). (Lower panel) Schematic illustrations of KC subtypes in a single MB calyx of each hymenopteran species. Class I “redefined” lKCs, mKCs, and “redefined” sKCs in the MB are colored green, magenta, and blue for the honeybee, scoliid wasp, and hornet, respectively. Two putative class I KC subtypes in the parasitic wasp MB are shown in yellow and light blue, respectively. The single putative class I KC subtype in the sawfly MB is shown in pale green. **(B)** Flowchart to produce homo-/heterozygous mutant workers by genome editing using CRISPR/Cas9. Mosaic queens (F0) with genome-edited germline cells are first produced by inducing fertilized embryos injected with sgRNA and Cas9 mRNA into queens. Subsequently, the mosaic queens are induced by transiently anesthetizing them with CO_2_ to lay unfertilized eggs, which grow into drones. Mutant drones (F1) derived from the mosaic queens are reared to adulthood, and the sperm collected from the sexually matured mutant drones is used to artificially inseminate a wild-type queen to produce a heterozygous queen (F2). Hetero- and homozygous mutant workers (F3) are produced by again artificially inseminating the heterozygous queen with sperm from the genome-edited drones. Figures for (A,B) are cited from Oya et al. (2017) and Kohno et al. (2016) with some modifications.

The brains of *V. mandarinia* and *C. prismatica* have three class I KC subtypes (lKCs, mKCs, and sKCs), as observed in the honeybee. In contrast, the brain of *A. reticulata* has only two KC subtypes; “large” KCs with significant *Trp*-expression and “small” KCs with no significant *Trp*-expression, and the brain of the sawfly *A. similis* has no discriminable KC subtypes (Farris and Schulmeister, 2011) (Figure 2A). Discrimination of class I and II KCs is difficult in *A. reticulata* and A. *similis*, because the MB calyces are shallow and Class I and II KCs seem to be merged in these species.

It is plausible that the advanced learning abilities of parasitic wasps to search for their host insects require MBs with elaborate calyces and both ancestral (original) and second KC subtypes, whereas the highly advanced learning abilities of aculeate insects to return to their nests require MBs with all of the class I KC subtypes, in addition to the elaborate MB calyces (Whitfield, 2003; Huber, 2009; Johnson et al., 2013). To test this notion, the correspondence between one and two KC subtypes detected in sawfly and parasitic wasps, and three KC subtypes detected in aculeate insects will need to be examined by *in situ* hybridization for homologs of genes expressed in a KC subtype-preferential manner in the honeybee (e.g., *Syt14, dlg5*, or *Mblk-1* for “redefined” lKCs; *mKas*t for mKCs; and *Trp* or *JHDK* for “redefined” lKCs/sKCs, respectively. See also Figure 1D) (Kubo, 2012; Kaneko et al., 2013, 2016; Suenami et al., 2016). The KC subtype/population that expresses FoxP in these hymenopteran insect species is also an intriguing topic for future investigation (Schatton and Scharff, 2017). Such experiments are expected to unveil KC subtype/population of ancestor origin in the hymenopteran insects and those unique to aculeate insects.

## Application of genome editing for analysis of the role of KC subtypes in the honeybee

While RNAi is effective for analyzing gene function, its efficiency sometimes varies depending on the animal species and target genes and/or organs (Matsumoto et al., 2014). In addition, it is difficult to suppress gene function for a long time (Matsumoto et al., 2014). An alternative method for the analysis of gene function is genome editing. Genome editing has been applied to some hymenopteran insects, including the sawfly *Athalia rosae* (Hatakeyama et al., 2016), parasitic wasp *Nasonia vitripennis* (Li et al., 2017), and two social ants, *Ooceraea biroi* and *Harpegnathos saltator* (Trible et al., 2017; Yan et al., 2017). A transgenic technique using *piggyBac* has been applied to honeybees (Schulte et al., 2014). Recently, Kohno et al. (2016) established a basic genome-editing technique in the honeybee to analyze *in vivo* gene function (Kohno et al., 2016).

To analyze the roles of genes in regulating the behaviors and/or brain functions exhibited by honeybee workers, it is necessary to produce hetero- or homozygous mutant workers (F3) through several steps (Figure 2B; Kohno et al., 2016). For this, it is important that adult mutant honeybees [mutant drones (F1) and homozygous mutant workers (F3)] should be alive; in other words, the target gene(s) must be dispensable for normal development and sexual maturation in honeybees. Kohno et al. selected *major royal jelly protein 1* (*mrjp1*) as a target gene to establish basic honeybee genome-editing techniques. The MRJP1 is the most abundant protein component of the royal jelly, which is produced by the hypopharyngeal glands of young nurse bees and secreted as food for the larvae, drones, and queens (Kubo et al., 1996; Ohashi et al., 1997; Schmitzová et al., 1998). As expected, the results indicated that *mrjp1* is dispensable for normal drone development (Kohno et al., 2016).

Genes expressed in a KC subtype-preferential manner can also be good candidate target genes for genome editing, because some of them are assumed to relate to some brain functions and some of them are dispensable for normal honeybee development and sexual maturation. Investigation of the functions of genes involved in development and sexual maturation will require other methods as well, such as the expression of knocked-in genes in a stage- and/or tissue-specific manner by genome-editing.

## Author contributions

TK drafted the manuscript and figures. SS, SO, HK, and TK wrote and reviewed the manuscript, and completed figures.

### Conflict of interest statement

The authors declare that the research was conducted in the absence of any commercial or financial relationships that could be construed as a potential conflict of interest.
